# Extracellular Vesicles and Post-Translational Protein Deimination Signatures in Mollusca—The Blue Mussel (*Mytilus edulis*), Soft Shell Clam (*Mya arenaria*), Eastern Oyster (*Crassostrea virginica*) and Atlantic Jacknife Clam (*Ensis leei*)

**DOI:** 10.3390/biology9120416

**Published:** 2020-11-25

**Authors:** Timothy J. Bowden, Igor Kraev, Sigrun Lange

**Affiliations:** 1 Aquaculture Research Institute, School of Food & Agriculture, University of Maine, Orono, ME 04469-5735, USA; timothy.bowden@maine.edu; 2 Electron Microscopy Suite, Faculty of Science, Technology, Engineering and Mathematics, Open University, Milton Keynes MK7 6AA, UK; igor.kraev@open.ac.uk; 3 Tissue Architecture and Regeneration Research Group, School of Life Sciences, University of Westminster, London W1W 6UW, UK

**Keywords:** protein deimination/citrullination, peptidylarginine deiminase (PAD), extracellular vesicles (EVs), immunity, metabolism, Mollusca, clam, oyster

## Abstract

**Simple Summary:**

Oysters and clams form an important component of the food chain and food security and are of considerable commercial value worldwide. They are affected by pollution and climate change, as well as a range of infections, some of which are opportunistic. For aquaculture purposes they are furthermore of great commercial value and changes in their immune responses can also serve as indicators of changes in ocean environments. Therefore, studies into understanding new factors in their immune systems may aid new biomarker discovery and are of considerable value. This study assessed new biomarkers relating to changes in protein function in four economically important marine molluscs, the blue mussel, soft shell clam, Eastern oyster, and Atlantic jacknife clam. These findings indicate novel regulatory mechanisms of important metabolic and immunology related pathways in these mollusks. The findings provide new understanding to how these pathways function in diverse ways in different animal species as well as aiding new biomarker discovery for Mollusca aquaculture.

**Abstract:**

Oysters and clams are important for food security and of commercial value worldwide. They are affected by anthropogenic changes and opportunistic pathogens and can be indicators of changes in ocean environments. Therefore, studies into biomarker discovery are of considerable value. This study aimed at assessing extracellular vesicle (EV) signatures and post-translational protein deimination profiles of hemolymph from four commercially valuable Mollusca species, the blue mussel (*Mytilus edulis*), soft shell clam (*Mya arenaria*), Eastern oyster (*Crassostrea virginica*), and Atlantic jacknife clam (*Ensis leei*). EVs form part of cellular communication by transporting protein and genetic cargo and play roles in immunity and host–pathogen interactions. Protein deimination is a post-translational modification caused by peptidylarginine deiminases (PADs), and can facilitate protein moonlighting in health and disease. The current study identified hemolymph-EV profiles in the four Mollusca species, revealing some species differences. Deiminated protein candidates differed in hemolymph between the species, with some common targets between all four species (e.g., histone H3 and H4, actin, and GAPDH), while other hits were species-specific; in blue mussel these included heavy metal binding protein, heat shock proteins 60 and 90, 2-phospho-D-glycerate hydrolyase, GTP cyclohydrolase feedback regulatory protein, sodium/potassium-transporting ATPase, and fibrinogen domain containing protein. In soft shell clam specific deimination hits included dynein, MCM3-associated protein, and SCRN. In Eastern oyster specific deimination hits included muscle LIM protein, beta-1,3-glucan-binding protein, myosin heavy chain, thaumatin-like protein, vWFA domain-containing protein, BTB domain-containing protein, amylase, and beta-catenin. Deiminated proteins specific to Atlantic jackknife clam included nacre c1q domain-containing protein and PDZ domain-containing protein In addition, some proteins were common as deiminated targets between two or three of the Bivalvia species under study (e.g., EP protein, C1q domain containing protein, histone H2B, tubulin, elongation factor 1-alpha, dominin, extracellular superoxide dismutase). Protein interaction network analysis for the deiminated protein hits revealed major pathways relevant for immunity and metabolism, providing novel insights into post-translational regulation via deimination. The study contributes to EV characterization in diverse taxa and understanding of roles for PAD-mediated regulation of immune and metabolic pathways throughout phylogeny.

## 1. Introduction

Molluscs represent one of the most important nonfed fishery products, whether sourced from the wild or from aquaculture. The Food and Agriculture Organization of the United Nations (FAO) estimates total global marine capture fisheries produced nearly 85 million tonnes in 2018 of which approximately 6 million tonnes (7.3%) was from molluscs [1]. When considering aquaculture products, molluscs constitute an even larger portion, making up over 21% of the total 82 million tonnes [1]. In the US, aquaculture production of oysters, mussels, and clams represents about 82% of the total value for marine aquaculture. Bivalve molluscs such as oysters, mussels, and clams form an important component of the food chain and food security and are of considerable commercial value worldwide. Furthermore, some are critical to ecosystem function and structure [2]. They are affected both by anthropogenic changes, such as water pollution and xenobiotics, as well as a range of pathogens including opportunistic pathogens also due to changes in sea climate. Furthermore, their immunity and metabolism is affected due to changes in ocean acidification and temperature [3]. Therefore, studies into their immune factors and associated biomarker discovery are of considerable value. The Mollusca species under study were the Eastern oyster (*Crassostrea virginica*), blue mussel (*Mytilus edulis*), soft shell clam (*Mya arenaria*), and Atlantic jacknife clam (*Ensis leei*).

The Eastern oyster (*C. virginica*) is a filter feeder which belongs to the class of Bivalvia, order Ostreida with important environmental value as it serves as a foundation species in marine environments of the western Atlantic estuaries, including through formation of oyster beds [4]. The Eastern oyster is of great commercial value and has steadily been declining due both to disease, mainly caused by protozoan parasites, and overfishing [5]. Furthermore, it is an important aquaculture species, especially for the East Coast of the USA.

The blue mussel (*M. edulis*), is a Bivalvia of the order Mytilida, native to the North Atlantic coast but also found on the French Atlantic coast and the British Isles [6]. Dense mussel populations form beds, which are important for their survival [7]. The blue mussel is a filter feeder with important roles in removing bacteria and toxins in estuaries, therefore making them important foundation species. Blue mussels have a range of marine predators as well as parasites, form an important part of the food chain, but have been in steady decline by 40% in the past 50 years [8]. 

The soft shell clam (*M. arenaria*) is a Bivalvia, order Myida, and a filter feeder with a number of predators. Its habitat ranges from the Western Atlantic Ocean north to Canada and south to the Southern states of the US [9]. They are furthermore found in the Eastern Atlantic Ocean, including the UK as well as in the North Sea. 

The Atlantic jacknife clam (*Ensis leei*) (also called razor clam or bamboo clam) is a burrowing Bivalvia in the order Adapedonta, family Pharidae [10]. It is mainly (and natively) found along the North Atlantic American coast, from South Carolina to Canada, while it is also found in Northern Europe [11], including the UK [10]. As it lives in deep, vertical, permanent burrows down to a water depth of 37–60 m [10,12], commercial fishing of razor clam is not very common, but dense subtidal razor clam beds have been exploited commercially [10,13]. The species currently supports a small fishery in NorthShore, MA [12] and is in the development phases for large scale aquaculture [14], wherefore studies into clam immunity may be of considerable interest [15]. The razor clam is very sensitive to environmental salinity and temperature changes, as well as to anthropogenic pollution, such as oil spills [10] and has a number of natural predators [16,17]. 

Due to the role of these Bivalvia in the ecosystem, as well as their commercial value, the identification of novel pathways in their immunity and metabolism is of considerable interest. As Mollusca lack adaptive immunity and have therefore evolved sophisticated innate immune defense strategies, they are also an interesting model for evolutionary studies on adaption of host–pathogen defense mechanisms [18]. Furthermore, in relation to ongoing studies in our laboratories on EV characterization and peptidylarginine deiminase (PAD) mediated protein deimination in the phylogenetic tree such a study in Mollusca is timely. 

PADs are a phylogenetically conserved calcium-dependent family of enzymes with multifaceted roles in health and disease. In mammals five PAD isozymes are known, while three PAD isozymes have been described in birds and reptiles, but only one PAD form in teleost and cartilaginous fish [19,20,21,22,23,24,25,26]. Furthermore, PAD homologues, also referred to as arginine deiminases (ADI) [27] have been described lower in phylogeny, including in parasites [28] and bacteria [29,30], as well as in fungi [31]. In Mollusca, PADs have though hitherto not been reported and no PAD/ADI homologues are present for Mollusca in NCBI or Swissprot databases. PADs convert arginine into citrulline in an irreversible manner, leading to post-translational modification (citrullination/deimination) in numerous target proteins of cytoplasmic, nuclear, and mitochondrial origin [19,21,22,23,24,25,26,32,33]. Deimination causes structural protein changes which can affect protein function and consequently downstream protein–protein interactions. Deimination can also contribute to neo-epitope generation, which results in inflammatory responses, as well as affect gene regulation via deimination of histones [34,35,36,37,38]. PADs are furthermore a key-driver of neutrophil extracellular trap formation (NETosis), a phylogenetically conserved antipathogenic mechanism [39,40,41]. As post-translational changes contribute to protein moonlighting, which allows one protein to exhibit different functions within one polypeptide chain [42,43], post-translational deimination may form part of a mechanism facilitating such functional diversity. Therefore, deimination mediated regulation of homologous and conserved proteins in the phylogenetic tree may provide information on the diversification of immune and metabolic pathway function throughout evolution. 

A majority of studies on PADs and downstream deimination have hitherto related to human pathological mechanisms, but recently a comparative body of research has focused on identifying putative roles for PADs in physiological and immunological pathways in a wide range of taxa throughout the phylogenetic tree, including land and sea mammals, reptiles, birds, bony, and cartilaginous fish, Myrostomata and Crustacea. In these studies, PADs have indeed been identified to have roles in mucosal, innate, and adaptive immunity in a range of taxa [21,22,23,24,25,26,44,45,46,47,48,49,50,51,52,53]. Importantly, PADs have also been identified as important players in infection and antipathogenic responses, including antiviral [54,55], antiparasitic [28], and antibacterial ones [29,30].

Extracellular vesicle (EV) biogenesis, and regulation of EV release from cells, has been found to be partly regulated by PADs and as this has been identified in a range of taxa, it appears to be a phylogenetically conserved function [28,30,56,57,58,59]. EVs participate in cellular communication and can be isolated from many body fluids, including serum and plasma. EVs play physiological and pathological roles via transfer of cargo proteins and genetic material, including in inflammatory responses, in infection and host–pathogen interactions [28,37,60,61,62,63,64]. Studies on EVs in comparative animal models are a growing field, including in sea animals such as on bony fish [51,52,53,65], cartilaginous fish [23], Arthropoda [49], and Crustacea [48,66], but research on EVs in Mollusca is still scarce. Recent studies have investigated roles for EVs (including EVs or outer membrane vesicles/OMVs released from bacteria) in Mollusca host–microbe interaction in symbiosis and during infection [67,68,69,70] and in mantle formation [71]. As EVs carry information from their cells of origin, their cargo signatures (including deiminated protein cargo) can be usable biomarkers [72,73], highlighting the need for expanding EV research across the phylogenetic tree, including in Mollusca.

The current study characterized EVs and assessed post-translational deiminated protein signatures in hemolymph of four Bivalvia Mollusca species. In this baseline study, deiminated proteins were assessed in total hemolymph to capture overall deiminated protein signatures, including those in hemolymph EVs. This study provides novel insights into Mollusca immunity and metabolism and adds to current understanding of the roles for post-translational modifications in functional diversification of conserved immune, gene regulatory, and metabolic proteins throughout phylogeny.

## 2. Materials and Methods

### 2.1. Hemolymph Sampling from Mollusca

Eastern oysters (*Crassostrea virginica*) were obtained from Pemaquid Oyster Company, Damariscotta, Maine, blue mussels (*Mytilus edulis*) were obtained from Hollander and Dekoning, Trenton, Maine, soft shell clam (*Mya arenaria*) were obtained from Downeast Institute, Beals, Maine, and Atlantic jacknife clam (*Ensis leei*) were collected from Beals Island, Maine (n = 4 per species). All species apart from the razor clams was sourced from licensed dealers. The razor clams were collected from the wild, within a designated zone. While not directly assessing the animals for pathology by diagnostics, any noticeable health issues and the harvest areas are regularly monitored for health status. Hemolymph, approximately 1 mL per animal, was collected using a 1 mL syringe and a 26 G needle from the foot muscle (soft shell clam and Atlantic razor clam) or adductor muscle (Eastern oyster and blue mussel). The hemolymph was then frozen at −80 °C until further use for the individual experiments.

### 2.2. Isolation of Extracellular Vesicles and Nanoparticle Tracking Analysis (NTA)

Mollusca EVs were prepared from the individual hemolymph (thawed on ice) of four animals per species, using sequential centrifugation and ultracentrifugation. Procedures were carried out according to previously standardized and described protocols [23,46,47,48], also following recommendations of MISEV2018 [74]. For each individual hemolymph-EV preparation, 100 μL of Mollusca hemolymph was diluted 1:5 in Dulbecco’s PBS (DPBS, ultrafiltered using a 0.22 μm filter, before use). This was then centrifuged for 30 min at 4000× *g* at 4 °C, to remove of apoptotic bodies and aggregates. Supernatants were then collected and ultra-centrifuged at 100,000× *g* at 4 °C for 1 h. This resulted in EV-enriched pellets, which were resuspended each in 500 µL DPBS and thereafter ultra-centrifuged again for 1 h at 100,000× *g*, at 4 °C. The final resulting EV pellets were resuspended each in 100 µL of DPBS and kept frozen at −80 °C until used in the procedures described below (all assessments where performed with EV preparations that had not been frozen for longer than 1 week). EV size distribution profiles were generated and EVs were quantified using nanoparticle tracking analysis (NTA), based on Brownian motion of particles in suspension, and carried out using the NanoSight NS300 system (Malvern Panalytical Ltd., Malvern, UK). Prior to NTA, the EV samples were diluted 1/100 in DPBS (10 μL of EV preparation diluted in 990 μL of DPBS). The diluted EV samples were measured on the NanoSight NS300, recording five repetitive reads, 60 s each. Particle numbers per frame were 40–60, camera settings were at level 12 for recording and for post-analysis the threshold was set at 3. Replicate histograms were generated from these videos using the NanoSight software 3.0 (Malvern), representing mean and confidence intervals of the five recordings for each sample.

### 2.3. Transmission Electron Microscopy (TEM)

Hemolymph EVs were further assessed for morphology using TEM. For each species a pool of EVs from four individual animals was assessed. The procedure was similar as previously described [24,46]. Following thawing of isolated EV pellets (stored frozen for 1 week before imaging), the EVs were resuspended in 100 mM sodium cacodylate buffer (pH 7.4). A drop (≈3–5 μL) of the EV suspension was placed onto a carbon film TEM grid, glow discharged beforehand. After 10–15 min of partially drying the EV suspension, the excess was removed by filter paper and the grid was placed onto a drop of a fixative solution (2.5% glutaraldehyde in 100 mM sodium cacodylate buffer (pH 7.0)) for 1 min at room temperature. Then the grid was washed by placing it in sequence onto three drops of distilled water, blotting excess of water by a filter paper. Finally, the sample was stained for 1 min with 2% aqueous Uranyl Acetate (Agar Scientific, Stansted, UK), stain excess was removed by filter paper, and the grid was left to dry before storing it. Imaging of EVs was carried out with a JEOL JEM 1400 transmission electron microscope (JEOL, Tokyo, Japan), at 80 kV accelerating voltage and 30,000× to 60,000× magnification. Digital images were recorded with an AMT XR60 CCD camera (Deben UK Ltd., Bury Saint Edmunds, UK).

### 2.4. Isolation of Deiminated Proteins in Mollusca Hemolymph–F95 Enrichment

Total deiminated proteins were isolated from a pool of hemolymph of the four different Mollusca species, respectively, using the F95 pan-deimination antibody (MABN328, Merck, Watford, UK) and the Catch and Release®v2.0 immunoprecipitation kit (Merck, UK). The F95-antibody specifically detects proteins modified by citrullination/deimination and has been developed against a deca-citrullinated peptide [75]. For each analysis, a pool of hemolymph from four individual animals (4 × 25 μL) per species was used for F95-enrichment, which was performed at 4 °C overnight, using a rotating platform. Elution of deiminated (F95-bound) proteins from the columns was performed according to the manufacturer’s instructions (Merck), and the protein eluate was thereafter diluted 1:1 in 2× Laemmli sample buffer (BioRad, Watford, UK). Samples were kept frozen at −20 °C until further use for SDS-PAGE analysis, Western blotting, and in-gel digestion for LC–MS/MS analysis, as described below.

### 2.5. Western Blotting Analysis

For Western blotting, SDS-PAGE was carried out on the hemolymph (a pool of hemolymph from four animals per species, respectively) of the four Mollusca under study, as well as the corresponding isolated EV samples (isolated from a corresponding hemolymph pool). All samples were diluted 1:1 in denaturing 2 × Laemmli sample buffer (containing 5% beta-mercaptoethanol, BioRad, UK) and heated for 5 min at 100 °C. Protein separation was carried out using 4–20% gradient TGX gels (BioRad UK), followed by Western blotting at 165 V for 1 h using a Trans-Blot® SD semi-dry transfer cell (BioRad, UK). Membranes were stained with PonceauS (Sigma Aldrich, Gillingham, UK) to assess even protein transfer and then blocked with 5% bovine serum albumin (BSA, Sigma, UK) in Tris buffered saline (TBS) containing 0.1% Tween20 (BioRad, UK; TBS-T) for 1 h at room temperature. Primary antibody incubation was carried out overnight at 4 °C on a shaking platform using the following antibodies for Mollusca sera: F95 pan-deimination antibody (MABN328, Merck; diluted 1/1000 in TBS-T) and anti-human PAD2 antibody (anti-PAD2, ab50257, Abcam, Cambridge, UK; diluted 1/1000), for detection of putative PAD protein homologues, due to PAD2 being the most conserved PAD isozyme and the anti-human PAD2 antibody was previously shown to cross-react with PADs across taxa [21,22,23,24,25,26,44,45,46,47,48,49,76,77]. For characterization of EVs isolated from the Mollusca sera, the phylogenetically conserved EV-marker CD63 (ab216130, Abcam, UK; diluted 1/1000), as well as Flotillin-1 (ab41927; diluted 1/1000) were used. The nitrocellulose membranes were washed following primary antibody incubation at RT in TBS-T for 3 × 10 min and thereafter incubated with HRP-conjugated secondary antibodies (anti-rabbit IgG (BioRad) or anti-mouse IgM (BioRad), respectively, diluted 1/3000 in TBS-T), for 1 h at RT. The membranes were washed for 5 × 10 min TBS-T and digitally visualized, using enhanced chemiluminescence (ECL, Amersham, Fisher Scientific UK, Loughborough, UK) in conjunction with the UVP BioDoc-ITTM System (Fisher Scientific, UK).

### 2.6. Silver Staining

SDS-PAGE (using 4–20% gradient TGX gels, BioRad, UK) was carried out under reducing conditions for the F95-enriched protein eluates from hemolymph of the four Mollusca, as described in Section 2.5 (derived from a pool of hemolymph from four individual animals per species). The gels were then silver stained according to the manufacturer’s instructions, using the BioRad Silver Stain Plus Kit (1610449, BioRad, UK).

### 2.7. LC–MS/MS (Liquid Chromatography with Tandem Mass Spectrometry) Analysis of F95 Enriched Proteins

Liquid chromatography with tandem mass spectrometry (LC–MS/MS) was carried out to identify deiminated protein candidates from hemolymph of the four Mollusca species under study (for each proteomic analysis a pool of n = 4 animals per species was used), according to previously described methods in other taxa [46,48,49]. LC–MS/MS analysis was carried out following in-gel digestion, with the F95-enriched protein preparations (diluted 1:1 in 2× Laemmli buffer and boiled for 5 min at 100 °C) run 0.5 cm into a 12% TGX gel (BioRad, UK). The concentrated protein band (containing the whole F95 eluate) was excised, trypsin digested and subjected to proteomic analysis using a Dionex Ultimate 3000 RSLC nanoUPLC (Thermo Fisher Scientific Inc, Waltham, MA, USA) system in conjunction with a QExactive Orbitrap mass spectrometer (Thermo Fisher Scientific Inc, Waltham, MA, USA), performed by Cambridge Proteomics (Cambridge, UK), as previously described [23,25,48,49]. The data was processed post-run, using Protein Discoverer (version 2.1., Thermo Scientific) and MS/MS data were converted to mgf files which were submitted to the Mascot search algorithm (Matrix Science, London, U.K.) to identify deiminated protein hits. Search for F95 enriched proteins from the four individual species was conducted against a common UniProt database against Mollusca (CCP_Mollusca_Mollusca_20201007, 405,520 sequences; 142,460,216 residues). An additional search was conducted against a common contaminant database (cRAP 20190401; 125 sequences; 41,129 residues). The fragment and peptide mass tolerances were set to 0.1 Da and 20 ppm, respectively, and the significance threshold value was set at of *p* < 0.05 and a peptide cut-off score of 41 was applied for the common Mollusca database (carried out by Cambridge Proteomics, Cambridge, UK).

### 2.8. Protein–Protein Interaction Network Analysis

To predict and identify putative protein–protein interaction networks associated to the deiminated proteins from Mollusca hemolymph, STRING analysis (Search Tool for the Retrieval of Interacting Genes/Proteins; https://string-db.org/) was performed. Protein networks were generated based on protein names and applying the function of “search multiple proteins” in STRING (https://string-db.org/). For a representative choice of database, California sea hare (*Aplysia californica*) was selected, as no species-specific protein databases are available for the four specific individual species under study in STRING. Networks were therefore built representative of the phylum Mollusca (with California sea hare showing most homology protein hits) and also compared with human networks, using the *Homo sapiens* STRING database, respectively. Parameters applied in STRING were as follows: “basic settings” and “medium confidence”. Color lines connecting the nodes represent the following evidence-based interactions for the network edges: “known interactions” (these are based on experimentally determined curated databases), “predicted interactions” (these are based on gene neighborhood, gene co-occurrence, gene fusion, via text mining, protein homology, or coexpression). Gene ontology network clusters for the deiminated protein networks were assessed in STRING and are highlighted by color coding (see the corresponding color code keys showing the individual nodes and connective lines within each figure; Figures 5–9).

### 2.9. Statistical Analysis

NTA curves were generated using the Nanosight 3.0 software (Malvern Panalytical Ltd., Malvern, UK). The NTA curves show mean (black line) and standard error of mean (SEM), and the confidence intervals are indicated (red line). Protein–protein interaction networks were generated using STRING (https://string-db.org/), applying basic settings and medium confidence. Significance was considered as *p* ≤ 0.05.

## 3. Results

### 3.1. Characterization of Mollusca Hemolymph–EVs

**Figure 1:**
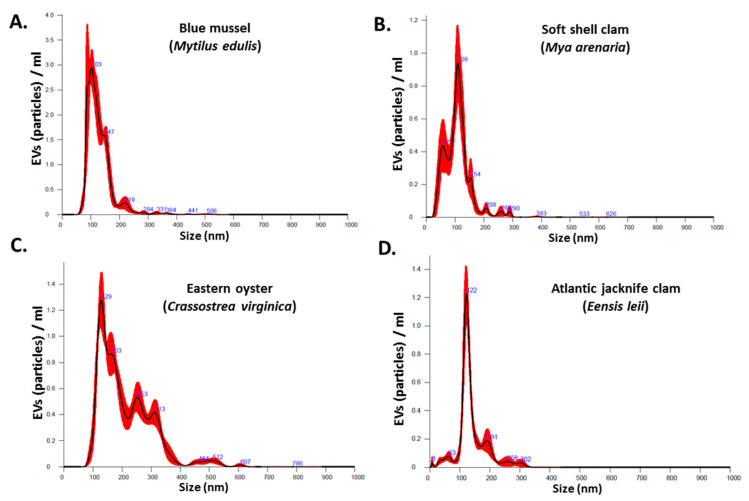
Nanoparticle tracking analysis (NTA) of Mollusca hemolymph EVs from (**A**) blue mussel; (**B**) soft shell clam; (**C**) Eastern oyster; (**D**) Atlantic jacknife clam.

The NanoSight NS300 was utilized for NTA assessment of particle numbers and size distribution of Mollusca hemolymph EVs. The EVs from the four different species were found to be poly-dispersed in the overall size range of 10–500 nm, with the majority of the EVs in the size range of 20–150 nm (Figure 1A–D). EV yield and EV modal size from the four different species under study showed some variability as follows: 

Blue mussel EV yield was 2.06 × 10^10^ particles/mL (SEM: ±1.91 × 10^9^ particles/mL) and modal EV size 102.8 ± 6.8 nm. Soft shell clam EV yield was 6.25 × 10^10^ particles/mL (SEM: ±4.48 × 10^9^ particles/mL) and modal EV size 115.6 ± 4.1 nm. Eastern oyster EV yield was 1.64 × 10^10^ particles/mL (SEM: ±6.42 × 10^8^ particles/mL) and modal EV size 126.2 ± 5.2 nm. Atlantic jacknife clam EV yield was 5.13 × 10^9^ particles/mL (SEM: ±3.27 × 10^8^ particles/mL) and modal EV size 123.0 ± 1.7 nm.

**Figure 2:**
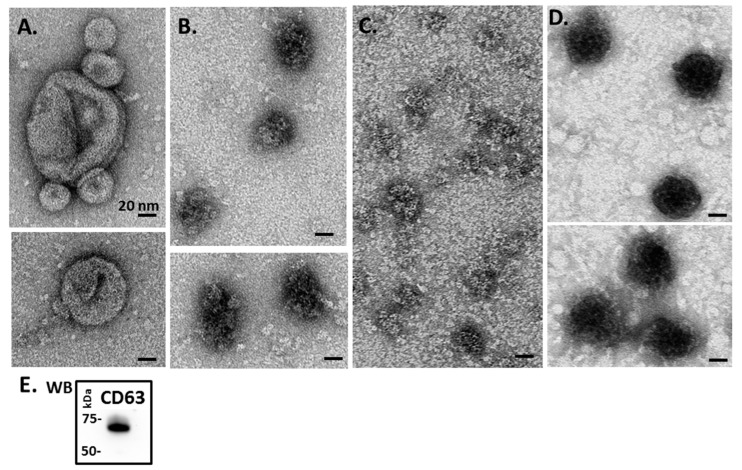
Transmission electron microscopy (TEM) analysis of Mollusca hemolymph EVs. (**A**) Blue mussel; (**B**) soft shell clam; (**C**) Eastern oyster; (**D**) Atlantic jacknife clam. (**E**) Western blotting (WB) of hemolymph EVs (representative figure showing EVs from soft Atlantic jacknife clam) shows strong CD63 positive (protein size standard is indicated in kilodaltons, kDa).

Transmission electron microscopy (TEM) revealed a majority of small EVs (“exosomes”; 20–100 nm sized) (Figure 2A–D), while some larger vesicles were also seen, particularly in blue mussel (Figure 2A) as well as in Eastern oyster (Figure 2C). Overall, TEM confirmed EV analysis observed by NTA. Assessment of EVs with the two phylogenetically conserved EV-specific markers CD63 and Flot-1, by Western blotting, showed strong positive reaction for CD63 (Figure 2E), which corresponds to the majority of vesicles being small EVs (“exosomes”), while Flot-1 did not show positive (not shown).

### 3.2. PAD Protein Homologue and Deiminated Proteins in Mollusca Hemolymph

**Figure 3:**
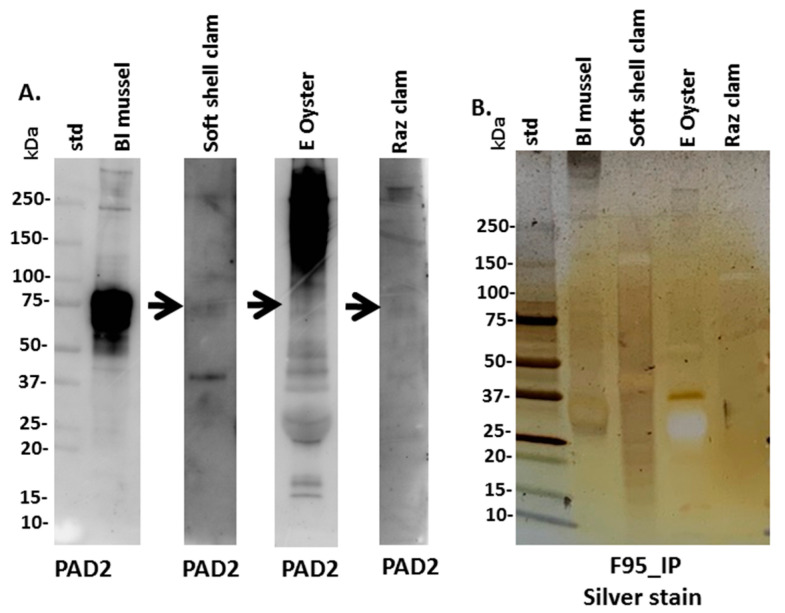
Mollusca PAD and deiminated proteins in hemolymph. (**A**) Western blotting analysis for PAD homologues in Mollusca, using the anti-human PAD2 antibody. (**B**) Silver stained SDS-PAGE gel (4–20% gradient TGX gel), showing F95-enriched fractions (F95_IP) from the four Mollusca species. All lanes show analysis of a pool from four individual animals, per species. The protein standard (std) is indicated in kilodaltons (kDa).

Anti-human PAD2 specific antibody was used for the assessment of a putative PAD protein homologue in Mollusca, based on cross-reaction, using Western blotting. A positive protein band at an expected approximate 70–75 kDa size was strongly identified in blue mussel, some faint reaction was seen in soft shell clam (see arrow in Figure 3A), while in Eastern oyster a reaction was seen at higher protein bands which looked unspecific, with a very faint reaction in the expected 70–75 kDa size (arrow in Figure 3A), and also some faint cross-reaction with a 70–75 kDa size band in Atlantic jacknife clam hemolymph (Figure 3A). To assess the presence of putative deiminated proteins in the Mollusca sera, F95-enriched fractions were separated by SDS-PAGE and silver stained, revealing protein bands in sizes ranging between 15 and 250 kDa (Figure 3B) and these were further subjected to proteomic (LC–MS/MS) analysis (Section 3.3).

### 3.3. LC–MS/MS Analysis of Deiminated Proteins in Mollusca Hemolymph

**Figure 4:**
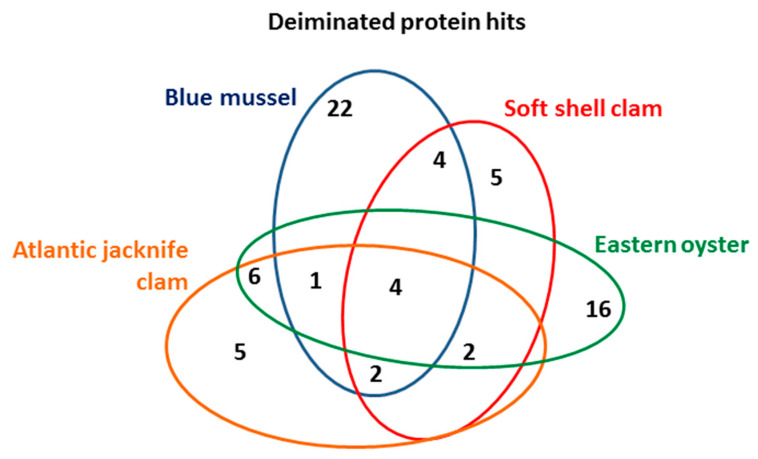
Deiminated protein hits in the four Mollusca species. The Venn diagram represents the number of deiminated proteins identified in and overlapping in blue mussel, soft shell clam, Eastern oyster, and Atlantic jacknife clam.

Deiminated protein identification of the Mollusca hemolymph (using a pool from four animals per species) was carried out following F95-enrichment using LC–MS/MS analysis. Species-specific protein hits with the individual species, as well as hits with other Mollusca were identified using the UniProt Mollusca database (Table 1, Table 2, Table 3 and Table 4; see Tables S1–S4 for full details on protein hits). Overall, 22 protein hits were specific to blue mussel, five hits were specific to soft shell clam only, 16 hits specific for Eastern oyster, and five protein hits specific for Atlantic jacknife clam. While these hits were found only in the individual species (using a pool of hemolymph from four animals per species), a number of further hits were shared between all or some of the species as outlined in Table 1, Table 2, Table 3 and Table 4 and the Venn diagram in Figure 4.

### 3.4. Protein–Protein Interaction Network Identification of Deiminated Proteins in Mollusca Hemolymph

**Figure 5:**
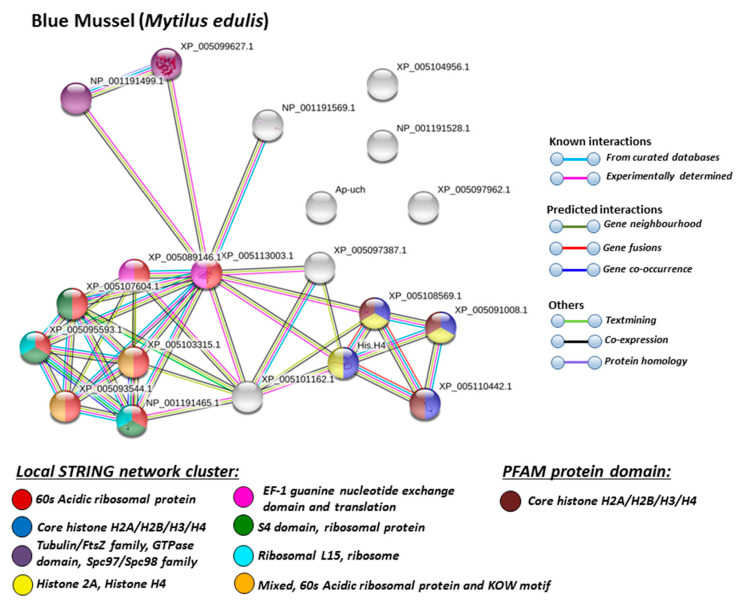
Local STRING network clusters and PFAM protein domains identified for deiminated proteins in blue mussel hemolymph. Protein–protein interaction network for blue mussel based on protein identifiers from Californian sea hare (*Aplysia californica*). PPI enrichment *p*-value: 0.000169. Color coding for network nodes and interaction lines is included in the figure.

**Figure 6:**
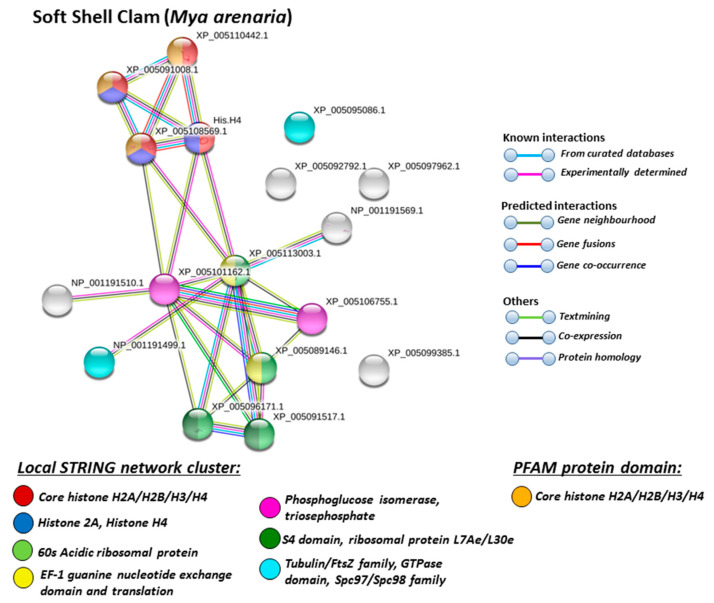
STRING network for soft shell clam. Protein–protein interaction network for soft shell clam based on protein identifiers in California sea hare (*Aplysia californica*). PPI enrichment *p*-value: 0.00477. Color coding for network nodes and interaction lines is included in the figure.

**Figure 7:**
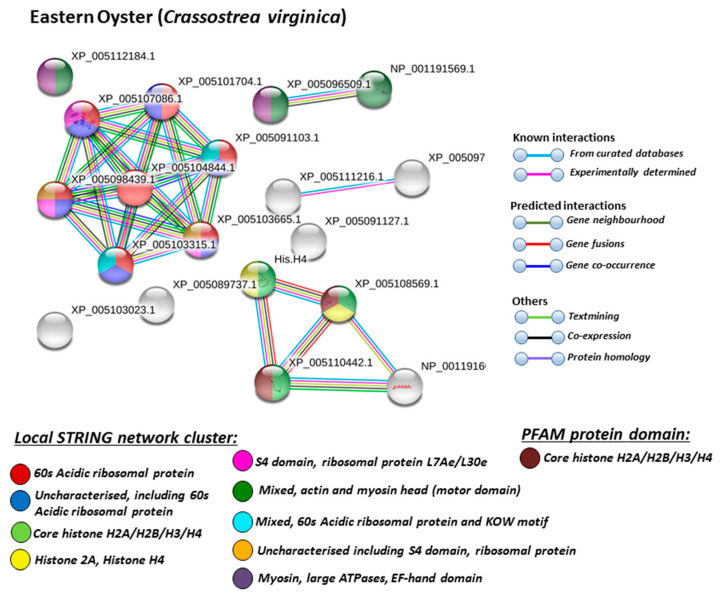
STRING network for Eastern oyster. Protein–protein interaction network for Eastern oyster, based on protein identifiers in California sea hare (*Aplysia californica*). PPI enrichment *p*-value: 0.254. Color coding for network nodes and interaction lines is included in the figure.

**Figure 8:**
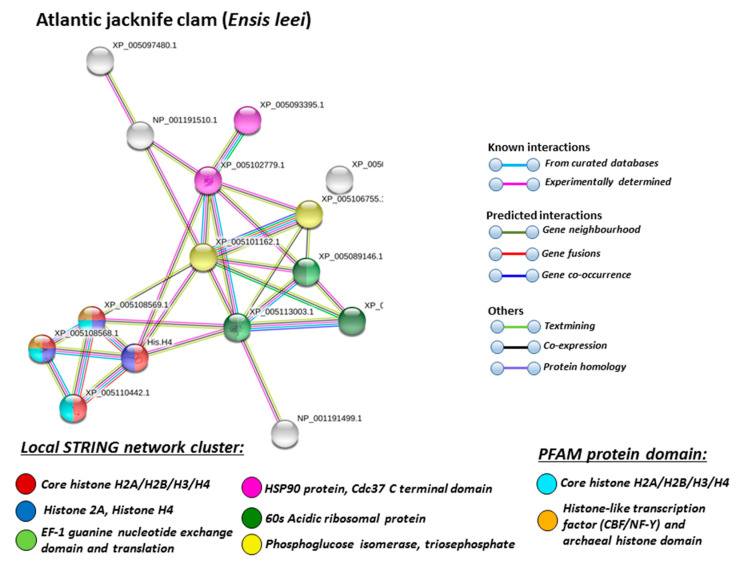
STRING network for Atlantic jacknife clam. Protein-protein interaction network for Atlantic jacknife clam, based on protein identifiers in California sea hare (*Aplysia californica*). PPI enrichment *p*-value: 0.0069. Color coding for network nodes and interaction lines is included in the figure.

**Figure 9:**
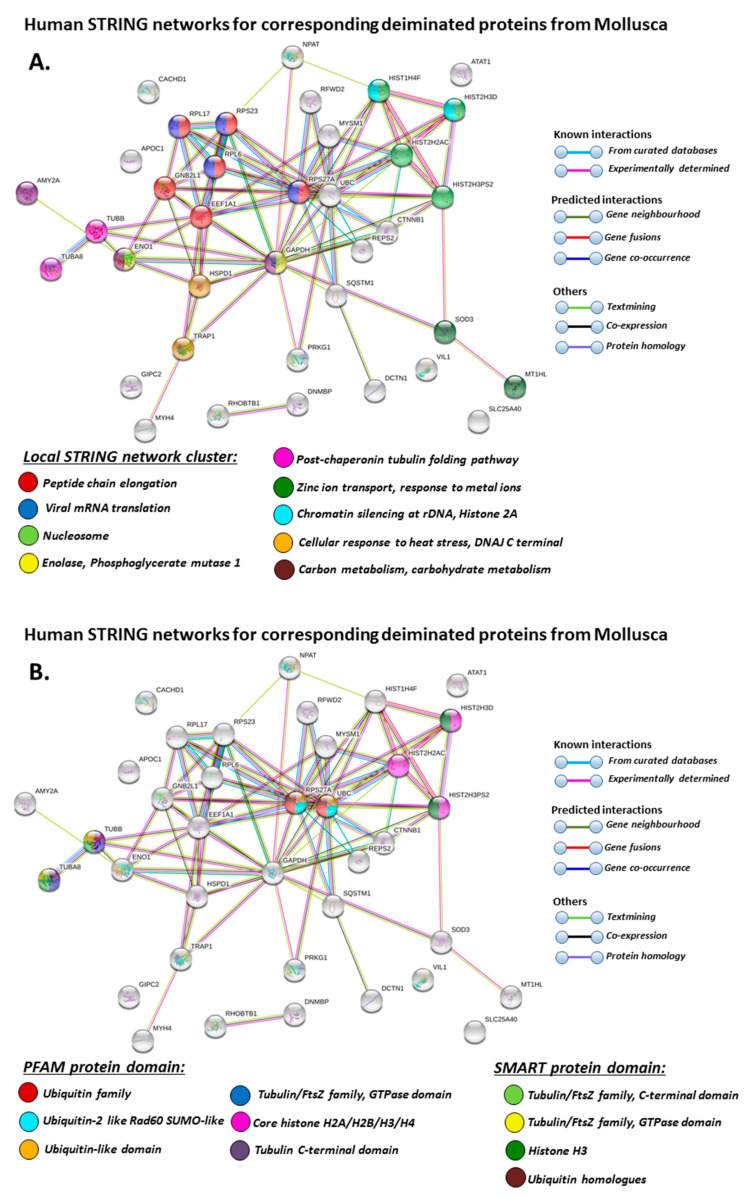

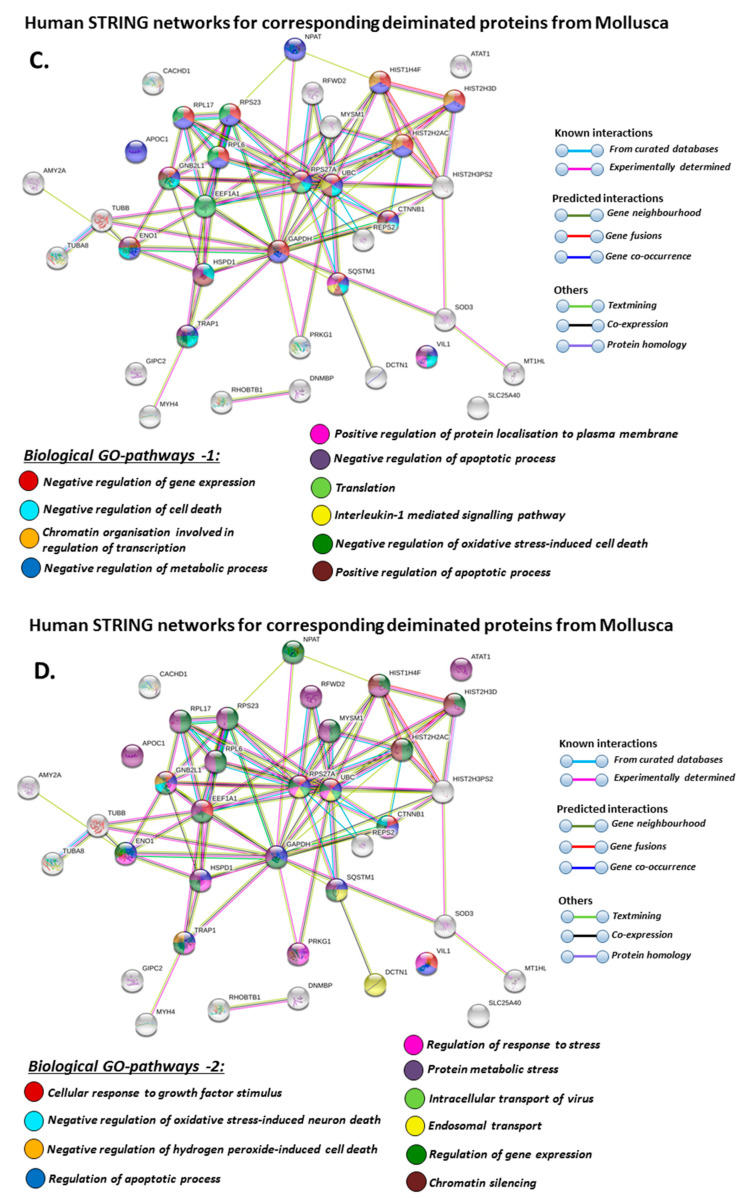

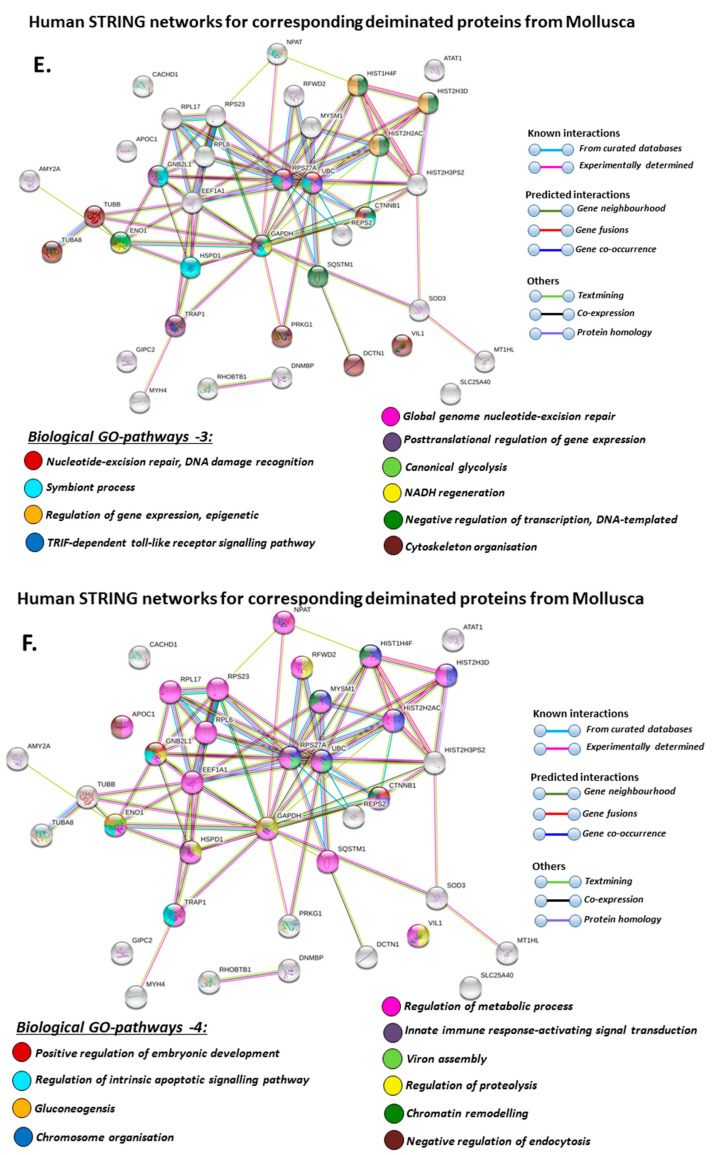
STRING protein interaction networks for deiminated protein hits identified in Mollusca, using human protein identifiers. (**A**) Local STRING network cluster; (**B**) PFAM and SMART protein domains; (**C**–**F**) biological GO processes: GO-1 (C), GO-2 (D), GO-3 (E), GO-4 (F). PPI enrichment *p*-value: 3.16 × 10^−8^. Color coding for network nodes and interaction lines is included in the figure.

For the prediction of protein–protein interaction networks of the deimination candidate proteins identified in the four different Mollusca species, the protein names were submitted to STRING (Search Tool for the Retrieval of Interacting Genes/Proteins) analysis (https://string-db.org/). Protein interaction networks were based on known and predicted interactions and represent all deiminated proteins identified in hemolymph of the different Mollusca species and their interaction partners present in the STRING database, based on networks for California sea hare (*Aplysia californica*) as a representative Mollusca species (this showed the maximum hits with the corresponding species-specific proteins identified by F95 enrichment in the four Bivalvia under study, although all proteins were not always found in the California sea hare database), as protein identifiers for the individual species was not available in STRING. The protein–protein interaction networks for each of the four Bivalvia species are represented in Figure 5, Figure 6, Figure 7 and Figure 8. In addition, STRING analysis was carried out for the whole list of F95-enriched hits identified in all the four Mollusca under study and protein interaction networks built based on corresponding human (*Homo sapiens*) protein identifiers (Figure 9).

Based on Mollusca protein interaction networks, the local STRING network clusters identified differed somewhat between the four species under study. Common networks between all four species were 60 s acidic ribosomal protein, S4 domain ribosomal protein, core histone H2A/H2B/H3/H4, tubulin/FtsZ family, GTPase domain, Spc97/Spc98 family, and EF-1 guanine nucleotide exchange domain and translation, 

In addition, blue mussel and Eastern oyster had enrichment for KOW motif; soft shell clam and Atlantic jacknife clam had enrichment for phosphoglucose isomerase triosephosphate; Atlantic jacknife clam had additional enrichment in HSP90 protein; Eastern oyster had enrichment for actin and myosin head (motor domain) as well as large ATPases. All four Mollusca species had PFAM protein domains enriched in deiminated proteins for core histone H2A/H2B/H3/H4, while Atlantic jacknife clam had furthermore deimination enrichment in histone-like transcription factor (CBF/NF-Y) and archaeal histone domain. 

When using corresponding human protein identifiers, additional enriched STRING network clusters for deiminated proteins included peptide chain elongation, viral mRNA translation, nucleosome, enolase, phosphoglycerate mutase 1, post-chaperoning tubulin folding pathway, zinc iron transport, response to metal ions, chromatin silencing at rDNA, Histone 2A, cellular response to heat stress, carbon and carbohydrate metabolism. PFAM protein domains enriched in deiminated proteins were ubiquitin related, in addition to tubulin and core histone ones. SMART protein domains related also to tubulin, histone, and ubiquitin. A large number of biological gene ontology (GO) pathways were furthermore identified based on human protein identifiers. This included regulation of gene expression and cell death, response to stress, interleukin signaling, TRI-dependent toll-like receptor signaling pathway, innate immune-response-activating signal transduction, intracellular transport of virus, viron assembly, regulation of proteolysis and endocytosis, cytoskeleton organization, chromatin organization, chromatin silencing, DNA damage recognition and nucleotide-excision repair, epigenetic and post-translational regulation of gene expression, regulation of metabolism, protein metabolic stress, canonical glycolysis, gluconeogenesis, NADH regeneration, as well as embryonic development regulation. 

## 4. Discussion

The current study characterized EVs and assessed putative PAD homologues and post-translational deiminated protein signatures in hemolymph of four Bivalvia species, providing novel insights into Mollusca gene regulatory processes, immunity, and metabolism while highlighting putative roles for post-translational modifications in functional diversification of conserved protein pathways throughout phylogeny. 

The EV profiles from the four different species showed some species-specific variation in size distribution, although overall the main peaks of EVs fell into a similar size range from 20 to 150 nm in all species. This also correlates with that the Mollusca hemolymph EVs showed strong positive for C63, a marker for small EVs (“exosomes”), and this has also previously been observed in EVs isolated from lobster and horseshoe crab hemolymph [48,49]. Research on EVs in Mollusca is a recent and growing field and previous studies have for example assessed the role for bacterial EVs (outer membrane vesicles/OMVs) in both host–pathogen interaction in Pacific oyster [67,69] as well as in symbiosis in bobtail squid (*Euprymna scolopes*) [68,70]. EVs from the Pacific oyster have also been assessed for microRNA content in response to bacterial infection, highlighting EVs as part of oyster immunity [69]. Therefore, EV profiling and further assessment of EVs in a wider range of Mollusca species, as in the current study, will be of considerable interest for investigation into both physiological and immune-related roles of EVs in Mollusca and for further development of associated cargo biomarkers. 

When building protein interaction networks for F95 enriched (deiminated/citrullinated) proteins for the four Bivalvia species under study, using the Mollusca database, most hits were found against the California sea hare (*Aplysia californica*), which was therefore used to create the protein networks and to identify pathways enriched in deiminated proteins. This analysis did underestimate the number of pathways affected by post-translational deimination as some protein identifiers, which varied between the four Mollusca species under study, were not present in the sea hare protein database. Therefore, further network analysis was also carried out based on corresponding human protein identifiers, revealing a considerable number of additional immune and metabolic related pathways to be enriched in deiminated proteins. 

The individual Mollusca protein hits identified to be deiminated showed some common targets between all four species (e.g., histone H3 and H4, actin, and GAPDH), while others were specific for the different species (e.g., heavy metal binding protein, heat shock proteins 60 and 90, sodium/potassium-transporting ATPase, fibrinogen domain-containing protein, muscle LIM protein, beta-1,3-glucan-binding protein, myosin heavy chain, thaumatin-like protein, vWFA domain-containing protein, BTB domain-containing protein, amylase), as discussed for the individual protein hits below. In addition, some proteins were common as deiminated targets between two or three of the Bivalvia species under study (e.g., EP protein, C1q domain containing protein, Histone H2B, tubulin, cold-shock domain protein, elongation factor 1-alpha, ubiquitin, dominin, and extracellular superoxide dismutase), further discussed below. These protein hits relate directly to the protein-networks identified in Figure 5, Figure 6, Figure 7, Figure 8 and Figure 9. They are discussed below in relation to the Mollusca literature, as well as in a more comparative context, where appropriate, for relevance of their function throughout phylogeny and therefore may provide some evolutionary insight into regulation of their function and protein moonlighting via deimination.

**Histones H2A, H2B, H3, and H4** were identified to be deiminated in the Mollusca in the current study and these are known deimination targets with roles in epigenetic regulation and antipathogenic responses in a range of taxa [23,24,25,26,47] as well as in relation to gene regulation in human pathologies, including cancer [37,78,79]. Histones serve as antimicrobial compounds in various species, ranging from crustaceans [80,81], amphibian [82], teleost fish [21,83], reptiles [84], pinnipeds and cetaceans [49,85], to human [86], including in mucosal immunity [87]. Histones have also been identified to have antimicrobial properties in Mollusca, for example H2A derived ones in disk abalone [88] and scallop [89], and histones H2B and H4 in oyster [90,91], where extracellular release of antimicrobial histones (extracellular trap formation/ETosis) is triggered by ROS [92,93]. Deimination/citrullination of histones in Mollusca is here though reported for the first time to our knowledge. Here, histone H2A was a deimination hit in soft shell clam and Atlantic jacknife clam, H2B was a deimination hit in blue mussel and eastern oyster, H3 and H4 were deimination hits in all four species. The regulation of multifaceted functions of histones via post-translational deimination requires further investigation throughout phylogeny, both in relation to physiological roles, including gene regulation and development, as well as antipathogenic and other immune responses.

**Actin** was a common deimination target in all four Mollusca under study. Actin is the major cytoskeletal protein in cells and both calcium and zinc have been shown to contribute to actin polymerization in oyster [94]. Actin cytoskeleton reorganization is also an important player in phagocytosis and has been assessed in *Vibrio* infection in the Pacific oyster (*Crassostrea gigas*) [95]. Actin filaments also control gene expression and chromatin remodeling complexes, which can be affected in oyster during heavy metal exposure [96]. Actin filaments are important for the transport of secretory vesicles, endosomes and mitochondria [97], and deimination may add to the multifaceted functions carried out by actins. Indeed, deimination of actin has been identified in a range of taxa, including Crustacea [48] and has also been directly associated with EV biogenesis in mammalian cells [56]. 

**Glyceraldehyde-3-phosphate dehydrogenase (GADPH)** was identified to be deiminated in hemolymph of all four Mollusca under study. It is an evolutionarily conserved enzyme [98] with key functions in the glycolytic pathway, as well as having roles in DNA repair, membrane fusion, and nuclear RNA export [99,100]. In oyster, GAPDH has been found to be reduced in response to pH and temperature changes, suggesting altered metabolism [3]. GAPDH has previously been identified as deiminated in teleost fish [21] and in lobster [48], pointing to a deimination-mediated regulatory role in its function. To what extent deimination contributes to GAPDH function in different taxa remains to be investigated.

**Heavy metal binding protein** was found deiminated in blue mussel, while EP protein, which also serves as a metal binding protein, was found deiminated in blue mussel and soft shell clam hemolymph. Invertebrates have naturally occurring heavy metal binding proteins, protecting the animals from excess uptake of metals and associated intoxication [101]. Indeed, even relatively low, but environmentally relevant, doses of metals such as manganese, lead, and cadmium can affect serotonin levels in mussels [102]. Furthermore, increase in toxic metal accumulation, including cadmium, caused by ocean acidification poses as a threat to a number of bivalve species [103]. Besides being a heavy metal detoxification protein, EP protein has been suggested to have multiple functions, acting also as a Ca^2+^ transport protein, as well as a shell matrix protein [104]. It may therefore be speculated that deimination, which is calcium-mediated, may mediate changes in protein structure and consequently protein function, facilitating protein moonlighting of EP.

**Cold-shock domain protein (CSDP)** was identified to be deiminated in blue mussel and soft shell clam. CSDPs form a group of evolutionarily conserved proteins with nucleic acid-binding ability, with multifaceted roles in cellular processes and are found in plants, bacteria, and animals. In Mollusca, they are involved in nutrient stress and adaptation to low temperature, including in cold stress responses [105,106]. The function of CSDPs is of major importance in relation to survivability under cold conditions in aquaculture, for example in the winter season, and has been studied in several species of clam and scallop [105,107]. The role of deimination has not been assessed in CSDP function and may, through structural and functional modification caused by this post-translational modification, add to their diverse functions across phylogeny.

**Heat shock proteins (HSP)** 60 and 90 were identified as deiminated in blue mussel. HSP60 is a multifunctional evolutionarily conserved protein with stress-protective roles in organisms [108]. HSP90 participates in the protein folding response, cell cycle control, organism development, and the proper regulation of cytosolic proteins and cell damage during stress, including thermal stress and bacterial challenge in oysters [109]. In Korean mussel (*Mytilus coruscus*), HSP90 has been found upregulated in response to *Vibrio* challenge, copper, cadmium, and 180 CST fuel exposure [110]. Interestingly, HSP has been found to be downregulated in gills of Hong Kong oysters (*Crassostrea hongkongensis*) exposed to long term heavy metal contaminated environments. This indicates significantly suppressed stress and immunity response systems of oysters in longer term toxin exposure, compared with shorter term [96]. In Manila clam, HSP60 was found to play roles in response to low-salinity and high-temperature stresses [108]. In freshwater clams both HSP60 and HSP90 overexpression was associated with high thermal tolerance [111]. Furthermore, HSP90 is associated with oxygen depletion stress in the Mediterranean mussel (*Mytilus galloprovincialis*) [112]. HSP has previously been reported as a deimination candidate in human pathologies in relation to rheumatoid arthritis, facilitating deimination-induced shifts in protein structure which aid B cell tolerance bypassing [113] and was also reported deiminated in llama serum under normal physiological conditions [24]. To what extent post-translational deimination of HSP plays a role in these various functions, including in Mollusca immune adaption to longer term exposure, remains to be assessed.

**Fibrinogen domain-containing protein** was found to be deiminated in blue mussel. In invertebrates, fibrinogen plays roles in immune defense, rather than roles in coagulation as is seen in higher animals [2,114]. Fibrinogen domain containing molecules have therefore ancestral roles in immunity—and they are highly polymorphic and diversified, possibly also allowing for anticipatory rather than adaptive immune responses [115]. Furthermore, through deimination, fibrinogen domains may acquire a range of roles throughout the phylogenetic tree, both as immune proteins as well as in coagulation pathways. In Mollusca, fibrinogen related proteins have been studied, where for example plasma lectins with fibrinogen motifs are involved in antiparasite responses of the snail *Biomphalaria glabrata* [115] and therefore may play roles in snail–Schistosoma host–pathogen compatibility [116]. In Eastern oyster (*Crassostrea virginica*), fibrinogen domain containing proteins have also been found to belong to immune-related gene families with high diversification and expression in response to bacterial challenge [117]. Indeed, fibrinogen is a known deimination candidate in humans, including in autoimmune disease [118,119] and has been identified as deiminated in a range of other taxa including reptiles and camelid [24,25]. This is the first report of deiminated fibrinogen in Mollusca and this modification may contribute to the multifaceted functions of fibrinogen across taxa, including host–parasite interactions.

**C1q domain containing protein (C1qDC)** was identified to be deiminated in blue mussel and Atlantic jacknife clam. C1qDC are homologues of vertebrate complement components, and are a diverse group of molecules that act as pathogen recognition receptors, also for a more specific responses to different pathogens [2]. This can be against a range of Gram-positive and Gram-negative bacteria, as well as fungi, as seen for different transcripts of C1qDC in mussels, clams, and scallop [120,121,122,123,124]. In Eastern oyster (*Crassostrea virginica*), C1q domain containing proteins are identified as immune-related gene families with high diversification and expression in response to bacterial challenge, similar as seen for fibrinogen domain containing proteins [117]. As functional confirmation on the observed C1qDC diversification is limited, it may be suggested that deimination could add to functional diversification throughout phylogeny, indeed as C1q has also been identified as a deimination target in mammals [26] and reptiles [25]. 

**VWFA domain-containing protein** was here identified as deiminated in Eastern oyster. vWF proteins have been described in a range of Mollusca. For example, vWF forms part of defense responses in the glue of terrestrial slug (*Arion subfuscus*), alongside C1q and lectin [125], and vWF domain is also found in other Mollusca defense proteins such as granularin [126]. Furthermore, vWF domain containing proteins have been found to participate in the biomineralization and formation of the nacre layer (mother of pearl) in Mollusca [127], a process which has been reported to require Ca^2+^-mediated protein–protein interactions [128]. Interestingly, deimination is such a process, as PAD/ADI driven citrullination/deimination is Ca^2^-mediated. Additionally, vWF domain containing proteins are involved in marine underwater adhesion through roles in load bearing and collagen manipulation to facilitate creation of a mussel’s holdfast [129], including during larval settlement in oyster [130]. Indeed, self-assembly of foot proteins has also been found to be a Ca^2+^-mediated process in pearl oysters [131]. Interestingly, in relation to nacre formation it has been reported that these domains have intrinsic disorder and cross-β-strand aggregation-prone regions [127], which theoretically makes them susceptible to post-translational deimination [32,132]. Indeed, vWF have previously been reported as deiminated in other taxa, for example in alligator [25], and deimination may therefore allow for moonlighting ability of vWF domain containing proteins throughout phylogeny.

**Muscle LIM protein** was found to be deiminated in Eastern oyster. It is involved in muscle development in vertebrates and regarded a key regulator of striated muscle physiology and pathophysiology in human. Furthermore, LIM-motif containing proteins play various roles in differentiation, cell fate, and cytoskeletal organization [133]. Interestingly, other diverse functions in immunity have also been identified in alternative taxa such as insects [134,135]. In Mollusca, changes in LIM expression is associated to immune and stress responses to *Vibrio* challenge in gills and digestive tract of the disk abalone (*Haliotis discus discus*) [136]. This is the first report of LIM to be deiminated and related structural and functional changes caused by this post-translational modification may have some effects on its diverse functions across taxa. It may furthermore be of relevance in relation to roles for LIM in myopathies and dystrophies, where LIM is involved in mechanotransduction and autophagy [137].

**Myosin heavy chain** was found to be deiminated in Eastern oyster. Myosin motors have, like actin, been found to be involved in the cellular transport of secretory vesicles, endosomes, and mitochondria [97]. Furthermore, myosin heavy chain has been studied in relation to metamorphosis on muscle development and remodeling in oyster larvae [138]. Myosin deimination may therefore contribute to its diverse biological moonlighting functions.

**Thaumatin-like protein** was identified as deiminated in Eastern oyster. It belongs to a superfamily of proteins, originally discovered in plants, involved in host defense and developmental processes in plants, fungi, and animals [139]. For example, they have wide-spectrum antifungal activities, including in animals such as nematodes and insects [140]. Thaumatin-like proteins contain lectin-like β-barrel motifs [140] and as beta-structures are prone to deimination [32,132], deimination of such motifs may be expected. The role for thaumatin in molluscan immunity has recently received attention following a proteogenomics analysis in the freshwater zebra mussel (*Dreissena polymorpha*) [141]. Deimination may add to the diverse functionality of this protein throughout phylogeny, including in Mollusca immunity.

**Ubiquitin** was identified as a deiminated protein hit in three of the Mollusca under study, blue mussel, Eastern oyster, and the Atlantic jacknife clam. Ubiquitin is phylogenetically conserved, causing post-translational ubiquitination in a range of proteins, which contributes to protein function diversity and plays important roles in physiological and pathological processes including homeostasis and vertebrate immune responses [142,143]. Ubiquitin can furthermore undergo post-translational modification itself [144], where for example methylation has been shown to affect cyclin degradation in clam embryo extracts [145]. Ubiquitin plays important roles in cellular homeostasis by regulation of autophagy, cellular damage, and stress [146]. Ubiquination has been studied in relation to innate immune responses of oyster and activation of inflammatory response in pathogenic infection [143]. Ubiquitin extracted from oyster gill has been shown to have antibacterial activity against Gram-positive and Gram-negative bacteria [147]. Antipathogenic pathways mediated by ubiquitin have also been identified in Crustacea [148,149]. Ubiquitin has furthermore been associated with cancer, neurodegenerative and autoimmune diseases [150,151,152], while in Mollusca, ubiquitin has been found to play roles in regulating synaptic strength and growth, using the sea slug (Aplysia) model [153]. In Mollusca, the ubiquitin-proteasome system has also been studied in relation to Schistosoma–snail pathogen–host interactions in *Biomphalaria glabrata* [154]. Deimination of ubiquitin was recently identified for the first time in Crustacea [48] and is here reported for the first time in Mollusca, indicating post-translational regulatory roles of ubiquitin related processes via deimination across different taxa.

A range of **ribosomal proteins** was identified in the blue mussel and Eastern oyster, and these relate amongst other to antibacterial effects [155], growth [156], and oxidative stress, including in response to trace metal exposure [157]. 

**Beta-1,3-glucan-binding protein (βGBPs)** was found deiminated in Eastern oyster. These play important roles as one group of soluble pattern recognition proteins in innate immune responses of invertebrates, binding to β-1,3-glucans from pathogens [2]. For example, in Chinese scallop βGBP is upregulated in response to *Vibrio* infection and identified as an inducible acute-phase protein [158]. In Asian green mussel (*Perna viridis*) βGBP has been identified to possess serine protease activity and agglutinating activity, pointing to more than one function for this protein [159]. In the Pacific oyster (*Crassostrea gigas*), multiple βGBPs have been identified with different immunological functions in circulatory hemocytes and digestive glands, respectively [160]. Peptides derived from βGBP of the Pacific abalone (*Haliotis discus hannai*) have furthermore been found to have both antimicrobial (antibacterial and antifungal) and antitumor activities against human cervix, lung, and colon carcinoma cell lines, by causing apoptotic cells death through disturbing cancer cell membranes [161]. The multifaceted functions of βGBPs may possibly be facilitated by protein deimination, and this will require further investigation.

**BTB domain-containing protein** was found deiminated in Eastern oyster. BTB-containing proteins are wide ranging and participate in many of cellular processes, including cell cycle regulation and actin dynamics, as well as having some shared function in recruitment of degradation targets to E3 ubiquitin ligase complexes. They also participate in diverse developmental processes [162], in cancer, musculoskeletal, and neurological diseases [163]. The high variability of BTB domain containing proteins furthermore facilitates different functional abilities of, for example, related transcription factors [164,165]. BTB have been associated to T-cell development, function, and regulation of T-cell mediated immunity [166] as well as metabolism and metabolic syndrome [167]. Furthermore, BTB-domain containing protein involvement in cancer has been identified via interaction with fibrinogen [168]. BTBs are also linked to splicing, transcriptional regulation, ion channel assembly and gating, stem cell regulation and in targeting proteins for ubiquitination [169,170]. In Mollusca, BTB have been studied in relation to voltage gated potassium channel formation [171]. BTB domain containing proteins are found throughout phylogeny and contain conserved beta-sheet structures in the core fold [169], indeed making them a strong deimination candidate. The deimination of BTB domain containing protein has not been reported before, and may be a newly identified mode for diversification of protein function and moonlighting. 

**Amylase** was identified to be deiminated in Eastern oyster. In humans and other mammals, amylase is found in the saliva, and it acts as a key digestive enzyme in phytophagous animals, playing roles in carbohydrate metabolism. Amylase is found throughout phylogeny from Archaea to mammals and has also been studied in a number of Mollusca, including oyster [172]. In Mollusca, amylase is related to growth rate, salinity and depending on species is expressed in digestive gland, digestive tract, hepatopancreas, and the mantle [173,174,175]. Amylase function can be modulated/inhibited via metals [176] and amylase is also involved in reproduction in oyster [177]. Amylase has also been associated with immune regulation and metabolic trade off under starvation in Bivalvia [178]. Amylase has not been reported to be deiminated in any species before to our knowledge and such post-translational modification may add to its functional diversity, also across phyla.

**Tubulin** was here identified as deiminated in blue mussel, soft shell clam, and Atlantic jacknife clam. Tubulin has roles in the rearrangement of the cytoskeleton and has been widely studied in Mollusca. For example, in surf clam in relation to cell division [179] and in relation to environmental stressors such as cadmium [180]. Tubulin has been identified as a deimination candidate in camelids [24] as well as in deiminated form being associated with EV biogenesis and release in mammalian cells [56]. Deimination of tubulin may therefore be crucial for facilitating diverse processes related to cytoskeletal rearrangement throughout phylogeny. 

**Elongation factor 1 alpha** was identified as a deimination hit in blue mussel, soft shell clam, and Atlantic jacknife clam. It has multiple roles in metabolic function, cell growth, cytoskeleton organization apoptosis, nuclear export of proteins, and the immune response [181,182,183,184]. It has been associated with stress tolerance in marine Mollusca species, including in response to copper exposure of early developmental (larval) stages of Chilean scallop (*Argopecten purpuratus*) [185]. Elongation factor 1 alpha has also been identified as a biomarker indicative of hypoxic stress, a problem for various marine species for example due to eutrophication, including the Mediterranean mussel (*Mytilus galloprovincialis*) [186]. Previously, it has been identified as a deimination candidate in teleosts [21] and in Crustacea [48]. The roles for deimination in multifaceted functions of elongation factor 1 alpha will need further exploration across taxa. 

**Extracellular superoxide dismutase (SOD)** was here identified to be deiminated in Eastern oyster. In human, superoxide dismutase is involved in oxidative stress [187] and various associated pathologies such as cancer, neurodegeneration, sarcopenia, and inflammatory diseases, but also longevity [188]. SOD has phylogenetically conserved roles in regulating oxidative stress resistance [189] and has furthermore been found to be affected by environmental xenobiotics [190]. In Mollusca, it has been related to reproductive investment and associated effects on antioxidant capacity in the gills of the Pacific oyster (*Crassostrea giga*), which is a species with a very high reproductive investment [191]. Deimination may contribute to the various function of SOD in different taxa.

**Dominin** was found deiminated in Eastern oyster and Atlantic jacknife clam. It is a major plasma glycoprotein, also associated with hemocytes, and has roles in host-defense and metal metabolism, as well as oyster shell formation [192,193,194]. Dominin has been identified as biomarker of oxidative stress due to being a target of protein thiol oxidation in relation to environmental contaminants [195]. Dominin is a multifaceted protein with diverse functions including sequestering metals to limit their availability to pathogens as part of host defense, has roles in antioxidation, as well as in wound repair [196]. Deimination is indeed linked to both hypoxic stress and wound healing [77,197]. The functional diversity of dominin may possibly be facilitated by post-translational modification, besides glycosylation, including by deimination reported here in the current study. 

In summary, this study provides an interspecific and descriptive comparison of deiminated protein profiles and EVs in four Bivalvia species, representative of the phylum Mollusca. It must be noted that the proteomic analysis was based on a pool of hemolymph from four animals per species to provide a baseline profile of deiminated proteins for that species, therefore not accounting for putative individual differences, which remain subject to further studies. EV profiles are described here for the first time in the blue mussel (*Mytilus edulis*), soft shell clam (*Mya arenaria*), Eastern oyster (*Crassostrea virginica*), and Atlantic jacknife clam (*Ensis leei*), showing some species specific size distribution profiles and dominance of CD63-positive vesicles. PAD homologues have here been identified for the first time in Mollusca based on cross-reaction by Western blotting using antibodies against human PAD2, considered as the phylogenetically most conserved PAD isozyme. Deiminated protein profiles in hemolymph of the four Bivalvia species indicate novel regulatory mechanisms via post-translational deimination of some major metabolic, immune related, and gene regulatory pathways, some of which are shared, also with other taxa, and others which seem species specific. It must furthermore be considered that some differences in deimination targets observed between the four species under study may be due to both possible biotic and abiotic factors at sampling, while the overall analysis of the four Bivalvia species does provide novel insights into deimination mediated pathways in Mollusca.

## 5. Conclusions

The analysis of deiminated proteins in several Mollusca species in this study indicates that protein deimination affects multiple Mollusca pathways involved in immunity and metabolism, as well as in gene regulation and this has previously been identified for PADs both in human disease as well as in diverse taxa, albeit with some species specific differences. Such post-translational regulation therefore may be a hitherto under-recognized conserved control-switch of immune and metabolic pathways throughout the phylogenetic tree, placing PADs in an interesting position as a “master” regulator in facilitating multifaceted protein functions via protein moonlighting. Furthermore, the characterization of EVs from four Mollusca Bivalvia indicates species-specific differences in EV profiles, opening a platform for further investigation into EV cargos and their roles in host–pathogen interactions, for the development of additional EV-related biomarkers in relation to the expanding global enterprise of Mollusca aquaculture.

## Figures and Tables

**Table 1:** Deiminated proteins in hemolymph of blue mussel (*Mytilus edulis*), as identified by F95-enrichment in conjunction with LC–MS/MS analysis. Deiminated proteins were isolated from hemolymph (a pool of hemolymph from four individual animals) by immunoprecipitation using the pan-deimination F95 antibody. The resulting F95-enriched eluate was then analyzed by LC–MS/MS and peak list files submitted to Mascot, using both a species-specific as well as a common Mollusca database. Peptide sequence hits are listed, showing species-specific hits, number of sequences for protein hits, and total score. Species hit names are indicated, blue mussel specific hits are on the top of the list and highlighted. *Proteins only identified in blue mussel. (See Table S1 for full details on all protein hits).

Protein ID *Protein Name*	*Species Name* Common Name	Matches (Sequences)	Total Score (*p* < 0.05) ^‡^
Q6UQ16_MYTED EP *protein*	*Mytilus edulis* Blue mussel	114 (6)	438
Q708T0_MYTED *Heavy metal binding protein	*Mytilus edulis* Blue mussel	40 (5)	291
Q05K66_MYTED *Actin (Fragment)*	*Mytilus edulis* Blue mussel	10 (3)	261
Q3S336_MYTED *Alpha-tubulin (Fragment)	*Mytilus edulis* Blue mussel	9 (3)	212
G0YFD6_MYTED *Tubulin beta chain (Fragment)	*Mytilus edulis* Blue mussel	7 (2)	201
A0A5P8PEH6_MYTED *Histone H4*	*Mytilus edulis* Blue mussel	6 (3)	131
Q9U9B5_MYTED *Actin (Fragment)*	*Mytilus edulis* Blue mussel	14 (2)	122
Q6WV83_MYTED *Histone H2B*	*Mytilus edulis* Blue mussel	4 (2)	78
A0A096ZTP0_MYTED *Histone H3*	*Mytilus edulis* Blue mussel	1 (1)	41
B0B039_MYTED *Ubiquitin*	*Mytilus edulis* Blue mussel	1 (1)	32
K1QG58_CRAGI Actin	*Crassostrea gigas* Pacific oyster	27 (8)	571
F0V443_MYTGA *Putative C1q domain containing protein MgC1q6	*Mytilus galloprovincialis* Mediterranean mussel	114 (6)	438
A0A077GY54_MYTTR *EP protein*	*Mytilus trossulus* Bay mussel	103 (6)	426
D3GA79_HALTU *Actin (Fragment)*	*Haliotis tuberculata coccinea* Green ormer	6 (5)	311
A0A2C9K042_BIOGL *Tubulin alpha chain*	*Biomphalaria glabrata* Freshwater snail	20 (4)	290
A0A433TJB9_ELYCH *Tubulin alpha chain*	*Elysia chlorotica* Eastern emerald elysia	15 (3)	215
V4A0D9_LOTGI *Histone H4*	*Lottia gigantea* Owl limpet	10 (5)	209
A0A076FGE1_PATRU *Tubulin beta chain	*Patella rustica* Rustic limpet	7 (2)	201
A0A6J8BQL5_MYTCO *TUBA	*Mytilus coruscus* Korean mussel	5 (3)	200
A0A649Z2S2_9EUPU *Actin (Fragment)*	*Hemphillia danielsi* Roundback slug	7 (6)	198
A0A077D3S6_MYTGA *Glyceraldehyde-3-phosphate dehydrogenase*	*Mytilus galloprovincialis* Mediterranean mussel	7 (2)	181
A0A649Z3D9_9EUPU *Actin (Fragment)*	*Hemphillia skadi* Skade’s jumping-slug	7 (5)	163
H6BD30_OSTED *GAPDH glyceraldehyde 3 phosphate dehydrogenase*	*Ostrea edulis* European flat oyster	15 (2)	136
A0A077GYT3_MYTTR *Cold-shock domain protein*	*Mytilus trossulus* Bay mussel	1 (1)	134
A0A3S0ZQE0_ELYCH *Uncharacterized protein (RAB1)	*Elysia chlorotica* Eastern emerald elysia	1 (1)	216
A0A6J8C382_MYTCO *H2A*	*Mytilus coruscus* Korean mussel	4 (3)	117
A0A4D6DEH0_9GAST *Histone H3 (Fragment)*	*Georissa similis* Land snail	6 (3)	111
E7DS85_9EUPU *Actin (Fragment)*	*Gulella pretiosa* Land snail	3 (3)	105
G3ET72_9BIVA *Tubulin beta chain	*Malletia johnsoni* Land snail	1 (1)	91
A0A6J7ZUB9_MYTCO *PGK*	*Mytilus coruscus* Korean mussel	1 (1)	82
A0A210QDC3_MIZYE *2-phospho-D-glycerate hydrolyase	*Mizuhopecten yessoensis* Yesso/Ezo scallop	2 (1)	81
A0A159WJ17_RUDPH *Heat shock protein 60	*Ruditapes philippinarum* Manila clam	1 (1)	78
A0A0B7ANE5_9EUPU *Tubulin beta chain	*Arion vulgaris* Spanish slug	1 (1)	75
A0A194ALQ1_PINFU *Elongation factor 1-alpha*	*Pinctada fucata* Akoya pearl oyster	1 (1)	70
V4AQU9_LOTGI *Tubulin_C domain-containing protein*	*Lottia gigantea* Owl limpet	11 (1)	64
K1RVE3_CRAGI *Actin*	*Crassostrea gigas* Pacific oyster	1 (1)	62
A0A077H3L5_MYTTR *40S ribosomal protein S27a	*Mytilus trossulus* Bay mussel	2 (2)	62
A0A0B7B879_9EUPU *Uncharacterized protein	*Arion vulgaris* Spanish slug	1 (1)	61
V4B3G5_LOTGI *Uncharacterized protein	*Lottia gigantea* Owl limpet	15 (1)	55
A0A499QNG2_RUDPH *Receptor for activated C kinase 1*	*Ruditapes philippinarum* Manila clam	6 (1)	51
K1PNQ5_CRAGI *Heat shock protein HSP 90-alpha 1	*Crassostrea gigas* Pacific oyster	1 (1)	51
A0A6J8AYS7_MYTCO *Uncharacterized protein	*Mytilus coruscus* Korean mussel	20 (1)	50
A0A6J8AKT9_MYTCO *Uncharacterized protein	*Mytilus coruscus* Korean mussel	7 (2)	50
A0A6J8AIA4_MYTCO *Uncharacterized protein (GTP cyclohydrolase 1 feedback regulatory protein)	*Mytilus coruscus* Korean mussel	2 (2)	49
A0A6J8B742_MYTCO *TRIM2_3	*Mytilus coruscus* Korean mussel	3 (1)	49
A0A0B7AV89_9EUPU *Sodium/potassium-transporting ATPase subunit alpha	*Arion vulgaris* Spanish slug	1 (1)	48
A0A433U913_ELYCH *Uncharacterized protein	*Elysia chlorotica* Eastern emerald elysia	1 (1)	48
A0A0L8FZD1_OCTBM *Uncharacterized protein	*Octopus bimaculoides* California two-spot octopus	2 (2)	43
A0A2T7NEC2_POMCA *Fibrinogen C-terminal domain-containing protein	*Pomacea canaliculata* Channeled applesnail	6 (1)	41
A0A3S1CEU4_ELYCH *Uncharacterized protein	*Elysia chlorotica* Eastern emerald elysia	5 (1)	41

^‡^ Ions score is −10*Log(P), where P is the probability that the observed match is a random event. Individual ions scores > 41 indicate identity or extensive homology (*p* < 0.05). Protein scores are derived from ions scores as a non-probabilistic basis for ranking protein hits.

**Table 2:** Deiminated proteins in hemolymph of soft shell clam (*Mya arenaria*), as identified by F95-enrichment in conjunction with LC–MS/MS analysis. Deiminated proteins were isolated from hemolymph (a pool of hemolymph from four individual animals) by immunoprecipitation using the pan-deimination F95 antibody. The resulting F95-enriched eluate was then analyzed by LC–MS/MS and peak list files submitted to Mascot, using both a species-specific as well as a common Mollusca database. Peptide sequence hits are listed, showing species-specific hit, number of sequences for protein hits, and total score. Species hit names are indicated, soft shell clam specific hits are on the top of the list and highlighted. * Proteins only identified in soft shell clam. (See Table S2 for full details on all protein hits).

Protein ID *Protein Name*	*Species Name* Common Name	Matches (Sequences)	Total Score (*p* < 0.05) ^‡^
V9VED0_MYAAR *Actin (Fragment)*	*Mya arenaria* Soft shell clam	7 (5)	255
Q6YNF3_MYAAR *Histone H3 (Fragment)*	Mya arenaria Soft shell clam	4 (2)	90
J9Z3Z3_MYAAR *Elongation factor 1 alpha *	*Mya arenaria* Soft shell clam	2 (1)	38
A0A0L8HIZ8_OCTBM *Uncharacterized protein (actin)*	*Octopus bimaculoides* California two-spot octopus	14 (7)	448
A0A6J8C382_MYTCO *H2A*	*Mytilus coruscus* Korean mussel	4 (2)	188
A0A6J8AIH4_MYTCO *H3*	*Mytilus coruscus* Korean mussel	6 (4)	164
A0A0B7B588_9EUPU *Tubulin alpha chain *	*Arion vulgaris* Spanish slug	2 (2)	150
A0A077D3S6_MYTGA *Glyceraldehyde-3-phosphate dehydrogenase*	*Mytilus galloprovincialis* Mediterranean mussel	3 (1)	140
V4A0D9_LOTGI *Histone H4*	*Lottia gigantean* Owl limpet	5 (2)	108
A0A6J7ZUB9_MYTCO *PGK*	*Mytilus coruscus* Korean mussel	2 (2)	94
A0A499QNG2_RUDPH *Receptor for activated C kinase*	*Ruditapes philippinarum* Manila clam	3 (1)	75
H6BD30_OSTED *GAPDH glyceraldehyde 3 phosphate dehydrogenase*	*Ostrea edulis* European flat oyster	8 (1)	71
A0A077GY54_MYTTR *EP protein*	*Mytilus trossulus* Bay mussel	1 (1)	68
A0A194ALQ1_PINFU *Elongation factor 1-alpha*	*Pinctada fucata* Akoya pearl oyster	3 (2)	65
A0A077GYT3_MYTTR *Cold-shock domain protein*	Mytilus trossulus Bay mussel	1 (1)	59
*A0A0R6BQX1_CRAHO* Superoxide dismutase	Crassostrea hongkongensis	1 (1)	56
K1QK39_CRAGI *Dynein heavy chain 2, axonemal	*Crassostrea gigas* Pacific oyster	7 (2)	56
V4AES5_LOTGI *Uncharacterized protein	*Lottia gigantean* Owl limpet	1 (1)	54
A0A2T7NYL0_POMCA *Uncharacterized protein*	*Pomacea canaliculata* Channeled applesnail	1 (1)	50
A0A6J8AYS7_MYTCO *Uncharacterized protein*	*Mytilus coruscus* Korean mussel	5 (1)	48
A0A210R3U3_MIZYE *80 kDa MCM3-associated protein	*Mizuhopecten yessoensis* Yesso/Ezo scallop	27 (2)	46
A0A6J8D0T9_MYTCO *PARP7S*	*Mytilus coruscus* Korean mussel	2 (1)	44
A0A6J8BH71_MYTCO *SCRN	*Mytilus coruscus* Korean mussel	1 (1)	42
A0A3S1AG64_ELYCH *Uncharacterized protein	*Elysia chlorotica* Eastern emerald elysia	20 (1)	41

^‡^ Ions score is −10*Log(P), where P is the probability that the observed match is a random event. Individual ions scores > 41 indicate identity or extensive homology (*p* < 0.05). Protein scores are derived from ions scores as a non-probabilistic basis for ranking protein hits.

**Table 3:** Deiminated proteins in hemolymph of Eastern oyster (*Crassostrea virginica*), as identified by F95-enrichment followed by LC–MS/MS analysis. Deiminated proteins were isolated from hemolymph (a pool of hemolymph from four individual animals) by immunoprecipitation using the pan-deimination F95 antibody. The resulting F95-enriched eluate was then analyzed by LC–MS/MS and peak list files submitted to Mascot, using both a species-specific as well as a common Mollusca database. Peptide sequence hits are listed, showing species-specific hit, number of sequences for protein hits, and total score. Species hit names are indicated, Eastern oyster specific hits are on the top of the list and highlighted. * Proteins only identified in Eastern oyster. (See Table S3 for full details on all protein hits).

Protein ID *Protein Name*	*Species Name* Common Name	Matches (Sequences)	Total Score (*p* < 0.05) ^‡^
Q0KJW4_CRAVI *Dominin*	*Crassostrea virginica* Eastern Oyster	47 (3)	293
H9ZXX0_CRAVI *Major plasma protein 2*	*Crassostrea virginica* Eastern Oyster	8 (4)	252
D9IA14_CRAVI *Histone H4*	*Crassostrea virginica* Eastern Oyster	6 (4)	241
Q92193|ACT_CRAVI *Actin (Fragment)*	*Crassostrea virginica* Eastern Oyster	3 (2)	149
A0A0C4URT1_CRAVI *Histone H3 (Fragment)*	*Crassostrea virginica* Eastern Oyster	1 (1)	41
A9XN85_CRAVI *Glyceraldehyde 3-phosphate dehydrogenase*	*Crassostrea virginica* Eastern Oyster	1 (1)	36
Q0KJW4_CRAVI *Dominin*	*Crassostrea virginica* Eastern Oyster	47 (3)	293
A0A6J8CKZ0_MYTCO *Uncharacterized protein (H4)*	*Mytilus coruscus* Korean mussel	7 (5)	282
H9ZXX0_CRAVI *Major plasma protein 2*	*Crassostrea virginica* Eastern Oyster	8 (4)	252
K1PY89_CRAGI *Extracellular superoxide dismutase [Cu-Zn]	*Crassostrea gigas* Pacific oyster	21 (2)	198
A0A0L8HIZ8_OCTBM *Uncharacterized protein (Actin)*	*Octopus bimaculoides* California two-spot octopus	8 (3)	192
A0A077D3S6_MYTGA *Glyceraldehyde-3-phosphate dehydrogenase*	*Mytilus galloprovincialis* Mediterranean mussel	3 (2)	143
K1R781_CRAGI *Muscle LIM protein Mlp84B	*Crassostrea gigas* Pacific oyster	3 (3)	128
K1RBZ0_CRAGI *Beta-1,3-glucan-binding protein	*Crassostrea gigas* Pacific oyster	4 (2)	111
K1RSS3_CRAGI *Myosin heavy chain, striated muscle	*Crassostrea gigas* Pacific oyster	3 (3)	103
K1QW36_CRAGI *60S ribosomal protein L6	*Crassostrea gigas* Pacific oyster	1 (1)	77
A0A0L8HPQ3_OCTBM *60S ribosomal protein L23	*Octopus bimaculoides* California two-spot octopus	1 (1)	72
K1QAH0_CRAGI *Thaumatin-like protein 1a	*Crassostrea gigas* Pacific oyster	2 (1)	72
A0A210QTG0_MIZYE *CEP209_CC5	*Mizuhopecten yessoensis* Yesso/Ezo scallop	6 (2)	62
A0A0B6Z082_9EUPU *40S ribosomal protein S23	*Arion vulgaris* Spanish slug	1 (1)	61
H6BD30_OSTED *GAPDH *	*Ostrea edulis* European flat oyster	5 (1)	61
A0A2T7NYP1_POMCA *Histone H2B*	*Pomacea canaliculata* Channeled applesnail	1 (1)	60
A0A076KW18_MYTGA *Ubiquitin C*	*Mytilus galloprovincialis* Mediterranean mussel	1 (1)	60
A0A0L8G4K0_OCTBM *VWFA domain-containing protein	*Octopus bimaculoides* California two-spot octopus	1 (1)	58
A0A2C9JK25_BIOGL *Uncharacterized protein	*Biomphalaria glabrata* Freshwater snail	4 (1)	56
A0A2T7NYL0_POMCA *Uncharacterized protein*	*Pomacea canaliculata* Channeled applesnail	2 (1)	55
K1QPG2_CRAGI *Uncharacterized protein	*Crassostrea gigas* Pacific oyster	1 (1)	51
A0A6J8AYS7_MYTCO *Uncharacterized protein*	*Mytilus coruscus* Korean mussel	2 (1)	48
V4BDC6_LOTGI *BTB domain-containing protein	*Lottia gigantean* Owl limpet	5 (2)	47
A0A0M7B3F1_9BIVA *Amylase	*Dreissena rostriformis bugensis* Quagga mussel	1 (1)	47
A0A3S1BNG5_ELYCH *RING-type domain-containing protein	*Elysia chlorotica* Eastern emerald elysia	1 (1)	44
A0A0L8HLW7_OCTBM *Uncharacterized protein	*Octopus bimaculoides* California two-spot octopus	1 (1)	44
A0A649Z2X2_9EUPU *Actin (Fragment)*	*Hemphillia skadi* Skade’s jumping-slug	3 (2)	44
A0A6J8D0T9_MYTCO *PARP7S*	*Mytilus coruscus* Korean mussel	2 (1)	42
A0A141BGR0_PINFU *Beta-catenin	*Pinctada fucata* Akoya pearl oyster	1 (1)	41
A0A210R431_MIZYE *Sequestosome-1*	*Mizuhopecten yessoensis* Yesso/Ezo scallop	1 (1)	41
K1QE77_CRAGI *Solute carrier family 25 member 40*	*Crassostrea gigas* Pacific oyster	1 (1)	41

^‡^ Ions score is −10*Log(P), where P is the probability that the observed match is a random event. Individual ions scores > 41 indicate identity or extensive homology (*p* < 0.05). Protein scores are derived from ions scores as a non-probabilistic basis for ranking protein hits.

**Table 4:** Deiminated proteins in hemolymph of Atlantic jacknife clam (*Ensis leei*), as identified by F95-enrichment followed by LC–MS/MS analysis. Deiminated proteins were isolated from hemolymph (a pool of hemolymph from four individual animals) by immunoprecipitation using the pan-deimination F95 antibody. The resulting F95-enriched eluate was then analyzed by LC–MS/MS and peak list files submitted to Mascot, using both a species-specific as well as a common Mollusca database. Peptide sequence hits are listed, showing species-specific hit, number of sequences for protein hits, and total score. * Proteins only identified in Atlantic jacknife clam. (See Table S4 for full details on all protein hits).

Protein ID *Protein Name*	*Species Name* Common Name	Matches (Sequences)	Total Score (*p* < 0.05) ^‡^
A0A6J8CKZ0_MYTCO *Histone H4*	*Mytilus coruscus* Korean mussel	9 (7)	314
A0A6J8C382_MYTCO *Histone H2A*	*Mytilus coruscus* Korean mussel	9 (7)	312
A0A1B1H1S7_9CAEN *Histone H3 (Fragment)*	*Fonscochlea zeidleri* Freshwater snail	6 (5)	200
V4A0D9_LOTGI *Histone H4*	*Lottia gigantea* Owl limpet	5 (4)	186
A0A076FHY5_PATCE *Beta-actin (Fragment)*	*Patella caerulea* Mediterranean limpet	5 (2)	170
A0A1V4JT39_PATFA *Glyceraldehyde-3-phosphate dehydrogenase*	*Mytilus galloprovincialis* Mediterranean mussel	2 (1)	111
A0A0K0PUN7_MYTGA *Nacre c1q domain-containing protein 1	*Mytilus galloprovincialis* Mediterranean mussel	2 (2)	101
Q0KJW4_CRAVI *Dominin*	*Crassostrea virginica* Eastern Oyster	4 (2)	85
A0A0R6BQX1_CRAHO *Superoxide dismutase*	*Crassostrea hongkongensis* Hong Kong Oyster	3 (1)	55
K1PY89_CRAGI *Extracellular superoxide dismutase*	*Crassostrea gigas* Pacific Oyster	3 (1)	55
H9ZXX0_CRAVI *Major plasma protein 2*	*Crassostrea virginica* Eastern Oyster	1 (1)	84
A0A6J7ZZ73_MYTCO *ACTB_G1	*Mytilus coruscus* Korean mussel	2 (2)	75
A0A1B2CWA8_FUSFL *Elongation factor 1-alpha*	*Fusconaia flava* Wabash pigtoe	2 (2)	72
H6BD30_OSTED *GAPDH glyceraldehyde 3 phosphate dehydrogenase*	*Ostrea edulis* European flat oyster	3 (1)	69
A0A6J8D0T9_MYTCO *PARP7S*	*Mytilus coruscus* Korean mussel	3 (1)	56
A0A2T7NYL0_POMCA *Uncharacterized protein*	*Pomacea canaliculata* Channeled applesnail	2 (1)	55
A0A0A7HG21_POMCA *Heat shock protein 90	*Pomacea canaliculata* Channeled applesnail	1 (1)	53
A0A0B7B588_9EUPU *Tubulin alpha chain*	*Arion vulgaris* Spanish slug	1 (1)	49
A0A6J8AYS7_MYTCO Uncharacterized protein	*Mytilus coruscus* Korean mussel	3 (1)	48
A0A076KW18_MYTGA *Ubiquitin C*	*Mytilus galloprovincialis* Mediterranean mussel	1 (1)	43
A0A210R431_MIZYE *Sequestosome-1*	*Mizuhopecten yessoensis* Yesso scallop	1 (1)	43
A0A0L8HUL5_OCTBM *PDZ domain-containing protein	*Octopus bimaculoides* California two-spot octopus	1 (1)	41
K1QE77_CRAGI *Solute carrier family 25 member 40*	*Crassostrea gigas* Pacific oyster	1 (1)	41
A0A6J8A7V5_MYTCO *Uncharacterized protein	*Mytilus coruscus* Korean mussel	3 (1)	40

^‡^ Ions score is −10*Log(P), where P is the probability that the observed match is a random event. Individual ions scores > 41 indicate identity or extensive homology (*p* < 0.05). Protein scores are derived from ions scores as a non-probabilistic basis for ranking protein hits.

## Supplementary Materials

Supplementary materials can be found at https://www.mdpi.com/2079-7737/9/12/416/s1. Full details on proteomic analysis of F95 enriched proteins can be found in Tables S1–S4 from: Table S1. Blue mussel (*Mytilus edulis*); Table S2. Soft shell clam (*Mya arenaria*); Table S3. Eastern Oyster (*Crassostrea virginica*); Table S4. Atlantic jacknife clam (*Ensis leei*).
